# Preparation and characterization of novel nanocombination of bovine lactoperoxidase with Dye Decolorizing and anti-bacterial activity

**DOI:** 10.1038/s41598-019-44961-2

**Published:** 2019-06-12

**Authors:** Esmail M. EL-Fakharany, Ahmed I. Abd-Elhamid, Nehal M. El-Deeb

**Affiliations:** 1 Protein Research Department, Genetic Engineering and Biotechnology Research Institute (GEBRI), City for Scientific Research and Technology Applications (SRTA-City), New Borg EL Arab, 21934 Alexandria Egypt; 2 Composites and Nanostructured Materials Research Department, Advanced Technology and New Materials Research Institute, City of Scientific Research and Technological Applications (SRTA-City), New Borg EL Arab, 21934 Alexandria Egypt; 3 Biopharmacetical Product Research Department, Genetic Engineering and Biotechnology Research Institute (GEBRI), City of Scientific Research and Technological Applications (SRTA-City), New Borg EL Arab, 21934 Alexandria Egypt

**Keywords:** Enzymes, Antimicrobials

## Abstract

Interaction between nanoparticles (NPs) and protein is particularly important due to the formation of dynamic nanoparticle-protein complex. The current study indicated that silica NPs were able to induce conformational modification in the adsorbed lactoperoxidase (LPO) which in turns degrades the synthetic dyes. The maximum degradation efficiency was recorded for the LPO modified silica NPs in the presence of H_2_O_2_ comparing with either free LPO or silica NPs. Degradation efficiency of crystal violet and commassie blue R250 after 6 h was assessed to be 100(%). Also, degradation efficiency of Congo red reached 90.6% and 79.3% in the presence and absence of H_2_O_2_, respectively, however methyl red degradation efficiency recorded 85%. The viability assay experiment indicated that the IC_50_ value of the LPO modified silica NPs on human fibroblast cells reached 2.8 mg/ml after 48 h incubation. In addition to dye removal, the LPO modified silica NPs were able to inhibit the antibiotic resistant bacterial strains (*Salmonell typhii*, *Staphylococcus areus*, *Pseudomonas aureginosa*, *E*. *coli*, *Proteus sp*. *and streptococcus sp*.) at concentrations up to 2.5 mg/ml with inhibition activity about 95%. These findings emphasized that the ability of LPO for degradation of the synthetic dyes after adsorption on silica NPs besides it could be a promising agent with potent inhibitory effect targeting a wide range of multidrug resistant bacteria.

## Introduction

Lactoperoxidase (LPO) is a glycoprotein with a molecular weight of about 78 kDa that having a covalently linked heme prosthetic group in its catalytic center. LPO was isolated from skimmed milk in crystalline state for the first time^1^ and later it was isolated from various exocrine secretions such as saliva, tears, and airways^2^. Moreover, there are other glands that are able to secrete this enzyme, such as lacrimal, Hardenian glands^3^ and salivary glands^4^. LPO belongs to a family of enzymes called peroxidases that their main function is primary control catalysis for the oxidation of certain specific molecules. All peroxidases use multiple steps to catalyse a similar oxidation reaction in the presence of hydrogen peroxide (H_2_O_2_) to generate various potent products with a wide biological and antimicrobial activities^5^. Initially, the antibacterial activity of LPO against lactic acid streptococci was suggested to be dependent on the existence of both H_2_O_2_, and thiocyanate ions (SCN−)^6^. Subsequently, various studies demonstrated that LPO mediates its antimicrobial activity through a specific inhibitory system named LPO system (LPS) involving LPO, SCNˉ and H_2_O_2_^7^. Additionally, LPS plays a significant role in the innate immunity system since the effect of LPS is not limited by the antimicrobial activity, while this system might play a potent role in the degradation of some toxins as aflatoxin^8^. The sensitivity of bacteria toward LPS varies among different strains. LPS was found to kill some catalase-positive bacteria, such as Gram-negative pseudomonas, salmonellae, shigellae, and coliforms bacteria in the proper environments, including specific temperature and pH of the medium, bacterial inoculum, and incubation period^6,9^. While LPS was found to inhibit but does not kill catalase-negative bacteria, such as Gram-positive streptococci and lactobacilli^10,11^. Moreover, LPS can significantly delay the growth of numerous psychrotrophic bacteria, such as several strains of the Micrococcus, Bacillus, and Pseudomonas genera^12–15^. This successfully increases the shelf life of food compared to what can be preserved with refrigeration only^9^.

Silica nanoparticles (NPs) are the common name for silicon dioxide (SiO_2_) which exists in crystalline and multiple amorphous forms. Silica NPs can be divided into natural silica and synthetic silica^16^. The synthetic silica NPs are widely used as additives, especially for cosmetics, drugs, food, and printer toners. The silica NPs can also be developed for use in biomedical and biotechnological applications such as cancer therapy, drug delivery, DNA transfection, protein coating, and enzyme immobilization^17–22^. Due to nano-size and large surface-to-mass ratio of NPs, they may interact with biological compounds such as proteins, nucleic acids, and lipids. Nanoparticle-protein corona (NP-PC) is formed as a result of interaction between the NPs and protein or adsorption of proteins on the NPs surface^22^. Adsorption of proteins on the NPs surface is happened by many forces such as Van der Waals interactions or hydrogen bonds. There are two types of corona; “hard corona” that binding between protein and NPs is irreversible and “soft corona” that binding between protein and NPs is rapidly reversible and have quicker separating rates^23–27^. The antibacterial activity of nano-metals has been recognized and the modification of their surface, to achieve new applications is growing fast^28,29^. Nano-metals have been used in dentistry for infection control and the management of the oral biofilm^30^. The antibacterial mechanism of nano-metals might include oxidative stress through the generation of activated oxygen species on the surface of NPs or by free metal ion toxicity. The activated oxygen species act by attacking bacterial polyunsaturated phospholipids and causing site-specific DNA damage^31,32^.

In the present study, we attempted to adsorb the purified LPO from bovine milk on the surface of silica NPs (SiO_2_) and applied for the degradation and decolorization of five synthetic dyes of Congo red, crystal violet, methylene blue, methyl red and commassie blue R250 compared to free silica NPs in the presence or absence of hydrogen peroxide as well as free LPO. We also evaluated the antimicrobial activity of this nanocombination against the common antibiotic resistant bacteria (*Salmonell typhii*, *Staphylococcus areus*, *Pseudomonas aureginosa*, *E*. *coli*, *Proteus sp*. *and streptococcus sp*.). NPs modify their physical and chemical features according to solid states, while the modification of their biological features is so far little recognized. For this purpose the biological harmlessness of these particles is essential, so it is necessary to assay its cytotoxic effect on the normal fibroblast cells. This novel modified NPs can be used in several applications such as in dentistry for bacterial infection control and in food industry for removing the toxic dyes and increasing the shelf life of food and dairy products.

## Results

### Purification of lactoperoxidase from bovine milk

**Figure 1:**
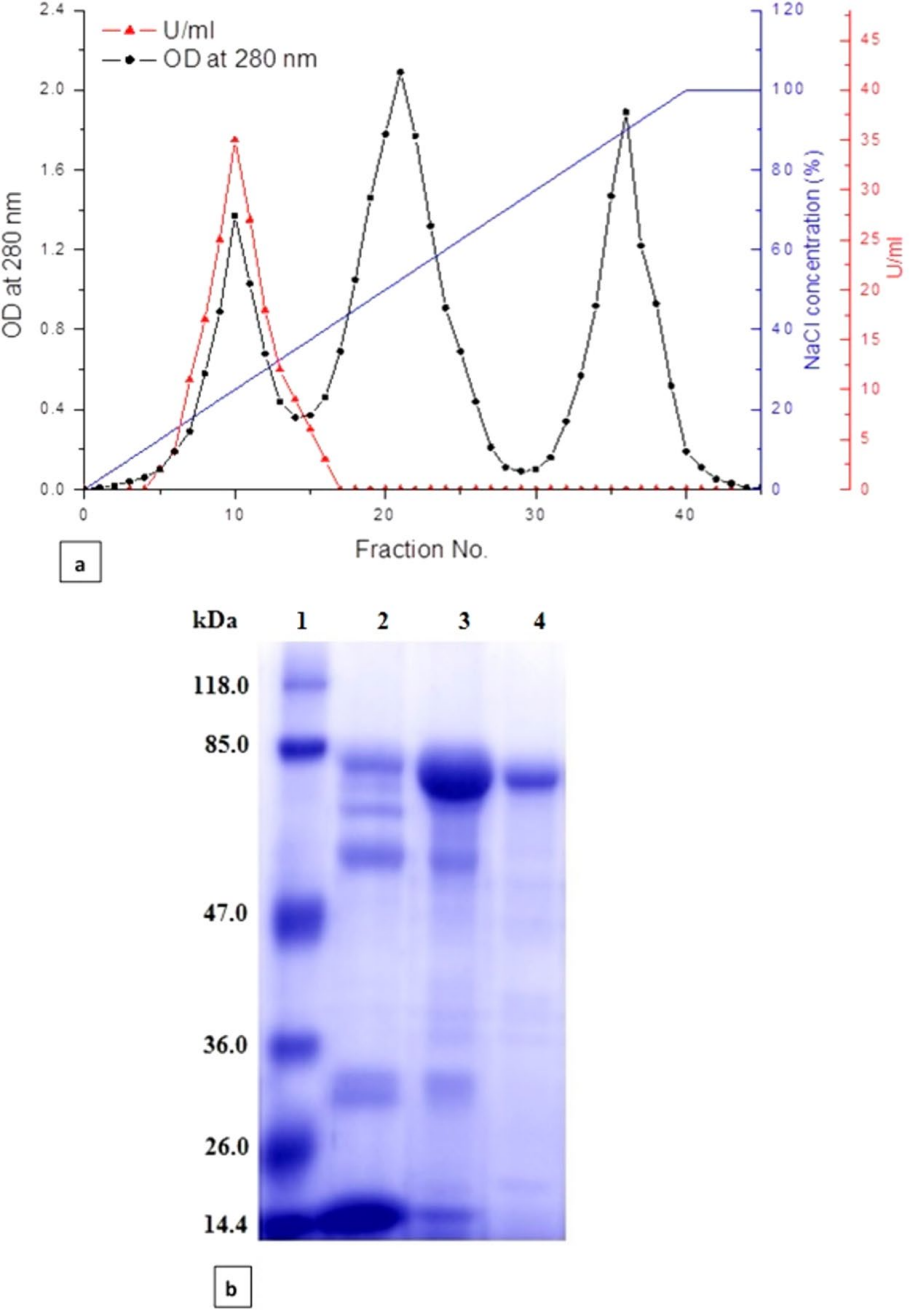
Purification and characterization of bovine LPO. (**a**) Elution profile of LPO and LF on CM-Sephadex C50 column. (**b**) 12% SDS-PAGE for LPO during purification steps. Lane 1 is protein marker, lane 2 is LPO eluted from CM Sephadex C50 column and lane 3 is purified LPO eluted from Sephacryl S100 column.

Lactoperoxidase (LPO) was isolated and purified from bovine milk after defatting and decaseination of milk. Firstly; skimmed milk was applied to cation exchange resin, CM-Sephadex C50 column equilibrated previously with 50 mM phosphate buffer, pH 7.0 then, LPO was eluted using a gradient of 0.0–1.0 M NaCl. The fractions containing LPO were assayed through measuring the activity of LPO using ABTS substrate (Fig. 1a). After pooling and dialysis of fractions containing LPO against 50 mM phosphate buffer, pH 7.0, the pooled sample was applied to Sephacryl S-100 column and LPO was eluted with a 50 mM phosphate, pH 7.0 containing 0.15 M NaCl. All Fractions that showed positive LPO activity were pooled, concentrated and desalted using centricon cut off 50 kDa. Using SDS polyacrylamide gel, LPO was detected at a molecular weight around 78 KDa as shown in Fig. 1b.

For further LPO purification conformations, Fig. S1 shows native zymogram of the purified LPO and skim milk. LPO enzyme was able to oxidize the guaiacol substrate in the presence of H_2_O_2_ to tetraguaiacol that form a yellowish band for active LPO on the zymogram. The specific activity of LPO was calculated for each step of purification and to the final purified enzyme solution as shown in Table 1. LPO was purified 3.56-fold from CM-Sepadex C50 column and 7.98-fold from Sephacryl S-100 column with a final yield of 45.6% and a specific activity of 45.5 unit/mg of LPO enzyme.

**Table 1:** Purification scheme for bovine lactoperoxidase.

Purification Step	Total protein (mg)	Total activity (units)*	S.A.(units/mg protein)	Fold purification	Recovery %
skimmed milk homogenate	210	1197	5.7	—	100
Mono S 5/50 GL column	40	812	20.3	3.56	67.8
Sephacryl S-100 column	12	546	45.5	7.98	45.6

^*^One unit of lactoperoxidase was defined as the increasing in the absorption as a result of the formation of the oxidized ABTS.

### Preparation and characterization of silica NP and the LPO modified silica NPs

**Figure 2:**
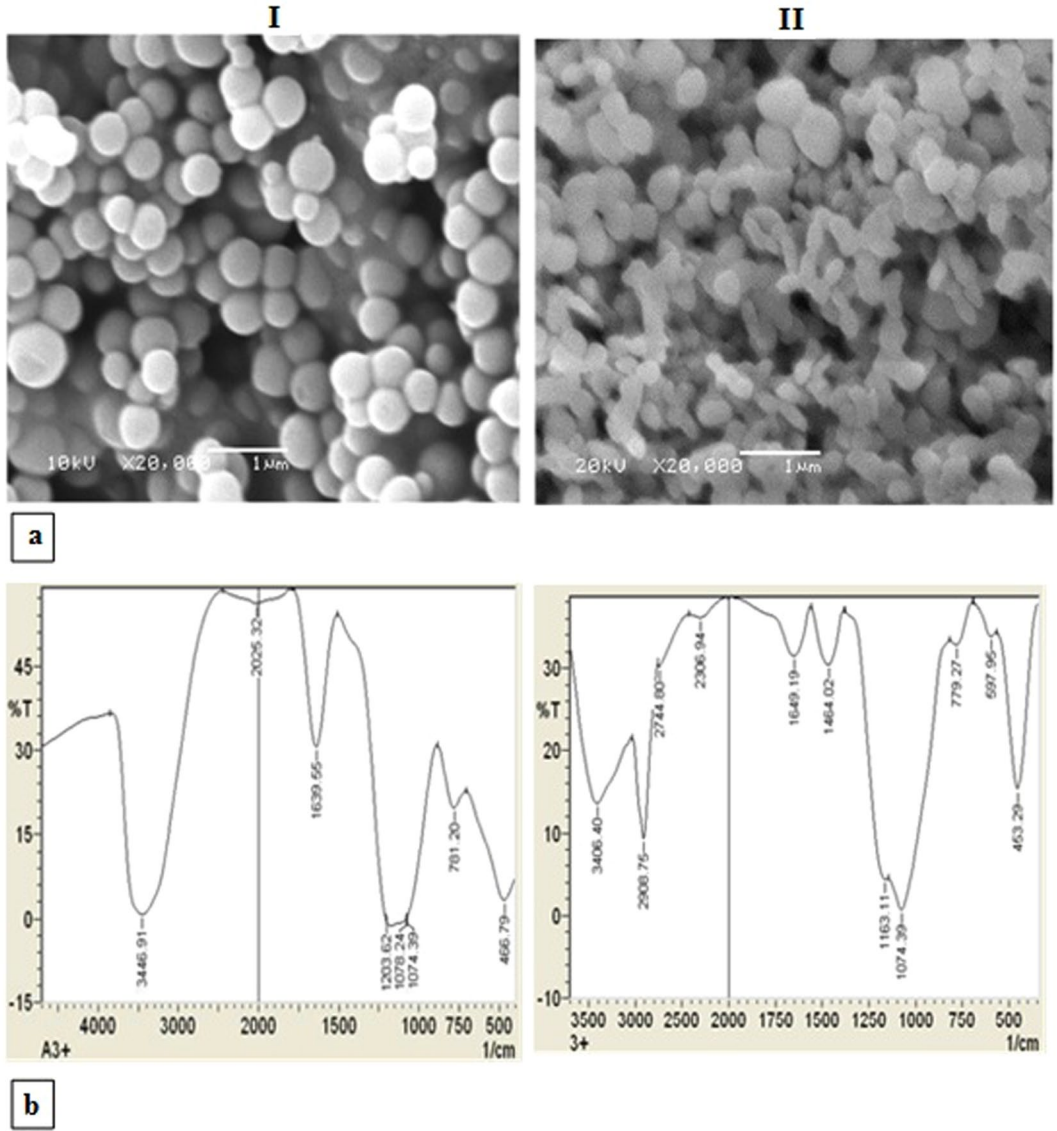
Scanning electron micrograph (**a**) and FTIR Spectra (**b**) of the prepared silica NPs (I) and the LPO modified silica NPs (II).

The particles size of the prepared silica NPs were detected by SEM. In Fig. 2a, the SEM picture showed homogenous particle size and uniformly distributed silica NPs with average particle size (0.449–0.639 µm). LPO was coated on silica NPs surface to form the LPO modified silica NPs with small size than silica NPs and the coating percentage of LPO on silica NPs was about 96%. The recovered silica NPs were formed in a well pattern and dispersed without aggregation after LPO coating (Fig. 2b).

Furthermore; the FTIR spectra of the recovered silica NPs shows common bands assigned to various vibrations in the solid (Fig. 2c). The analysis of these spectra revealed the broad-band centered at around 3446 cm^−1^ corresponding to the overlapping of the O-H stretching bands of hydrogen-bonded water molecules (H-O-H…H) and SiO-H that showed a stretching of surface silanols hydrogen-bonded to molecular water (SiO-H…H_2_O). The absorption bands corresponding to the adsorbed water molecules deformation vibrations appear at 1639 cm^−1^. The very intense and broad-band appearing at 1074 cm^−1^ and the shoulder one was assigned around 1203 cm^−1^, respectively to the transversal optical (TO) and longitudinal optical (LO) modes of the Si-O-Si asymmetric stretching vibrations. On the other hand, the symmetric stretching vibrations of Si-O-Si appear at 781 cm^−1^ and its bending mode appears at 466 cm^−1^. Additionally, Fig. 2d shows the FTIR spectra of the LPO modified silica NPs that showed peaks characterized SiO_2_ at 3446, 1639, 1203, 781 and 466 cm^−1^ with sifting to 3406, 1649, 1163, 779 and 453 cm^−1^, respectively. Moreover, another two peaks were noticed, the first peak at 1464 cm^−1^ corresponding to C-H vibration and this peak was easily assigned to the C-H symmetric deformation of -CH_2_ groups, and the second peak with strong sharp intense at 2908 cm^−1^ corresponding to symmetric stretching of C-H group. This is due to the interaction between the protein and SiO2 NPs.

### Degradation of synthetic dyes by the LPO modified silica NPs

**Figure 3:**
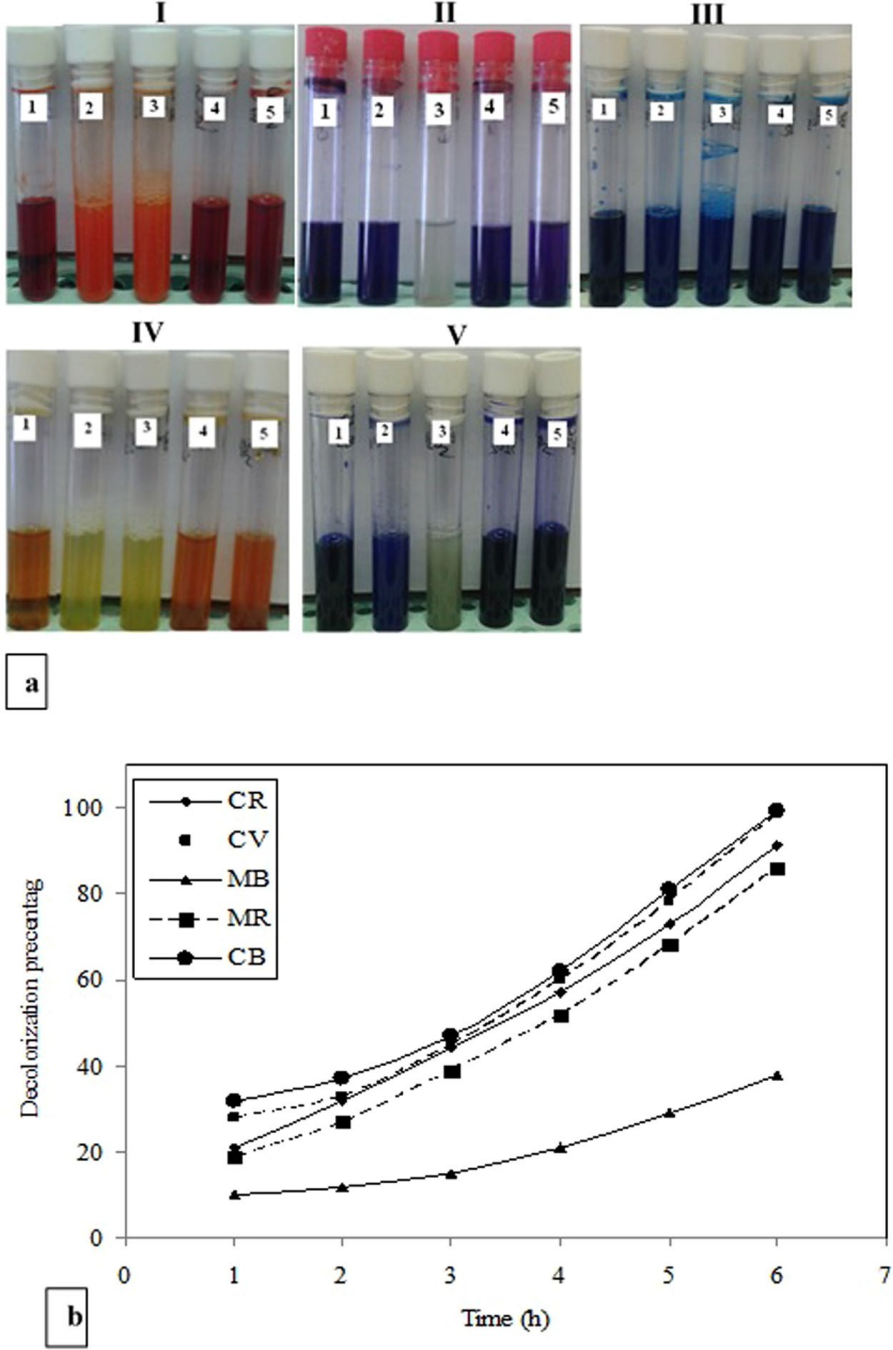
Decolorization efficiency of the LPO modified silica NPs. (**a**) Photographs of the decolorization samples of Congo red (I), crystal violet (II), methylene blue (III), methyle red (IV) and commassie blue R250 (V) at concentration of 500 mg/L at pH 7.0 as control (1) by the LPO modified silica NPs (2.5 mg/l) in the presence or absence of H_2_O_2_ (2 and 3) and by silica NPs (2.5 mg/l) in presence or absence of H_2_O_2_ (4 and 5). (**b**) Decolorization percentages of Congo red (CR), crystal violet (CV), methylene blue (MB), methyle red (MR) and commassie blue R250 (CB) at concentration of 500 mg/L by the LPO modified silica NPs (2.5 mg/l) in presence of 3.2 mM H_2_O_2_.

The obtained data in Fig. 3a and Table 2 show the ability of both LPO modified silica NPs and silica NPs in decolorization and degradation of Congo red, crystal violet, methylene blue, methyl red and commassie blue R250 dyes (at concentration of 500 mg/l) with and without hydrogen peroxide comparing with the negative control of each dye sample. From these results, it can be concluded that the complete degradation of crystal violet and commassie blue R250 was achieved after 6 h by using the LPO modified silica NPs in the presence of H_2_O_2_ while; in the absence of H_2_O_2_, this degradation percent decreased to be around 50%. The degradation efficiency for Congo red was 90.6% and 79.3% using the LPO modified silica NPs in the presence or absence of H_2_O_2_, respectively. Whereas, the degradation efficiency of methyl red was around 85% in the presence or absence of H_2_O_2_. Also, the degradation efficiency of methylene blue reached 37.7% and 20.9% only by using the LPO modified silica NPs in the presence and absence of H_2_O_2_, respectively.

**Table 2:** Decolorization percentage of synthetic dyes using 5 mg/ml of coated silica NP by LPO (LPO-SiO_2_) and silica NP (SiO_2_) before and after adding 50 µl H_2_O_2_ after 6 h.

	LPO-SiO_2_ (%)^*^	LPO-SiO_2_ + H_2_O_2_ (%)	SiO_2_ (%)	SiO_2_ + H_2_O_2_ (%)
Congo red	79.3 ± 0.3	90.6 ± 0.21	3.5 ± 0.9	11.4 ± 0.08
Crystal violet	44.4 ± 0.5	98.4 ± 0.94	14 ± 0.11	17.7 ± 0.32
Methylene blue	20.9 ± 0.31	37.7 ± 0.53	18.4 ± 0.4	23.76 ± 0.64
Methyl red	85.5 ± 0.24	85.8 ± 0.34	5.5 ± 0.12	4.5 ± 0.23
Comassie blue R250	51.5 ± 0.43	99 ± 0.45	1 ± 0.01	7 ± 0.12

^*^Synthetic dyes without decolorization at concentrations of 500 mg/l were used as negative control (0.0%). All values were expressed as mean ± SEM.

Furthermore, our results indicated that the degradation efficiency of silica NPs to Congo red, methylene blue, methyl red and commassie blue R250 in the presence or absence of H_2_O_2_ showed low degradation efficiency and reached 2–10%, while crystal violet degradation efficiency reached 17.7%. Also, methylene blue showed degradation efficiency reached 23% using silica NPs. The rate of decolorization and degradation percentages of Congo red, crystal violet, methylene blue, methyl red and commassie blue R250 synthetic dyes using the modified silica NPs in the presence of H_2_O_2_ was indicated in Fig. 3b. The decolorization dye adsorption rates started with low efficiency at the first hour and increased steadily over time to reach their maximum decolorizing capacity after 6 h of incubation.

### Infrared spectral analysis

**Figure 4:**
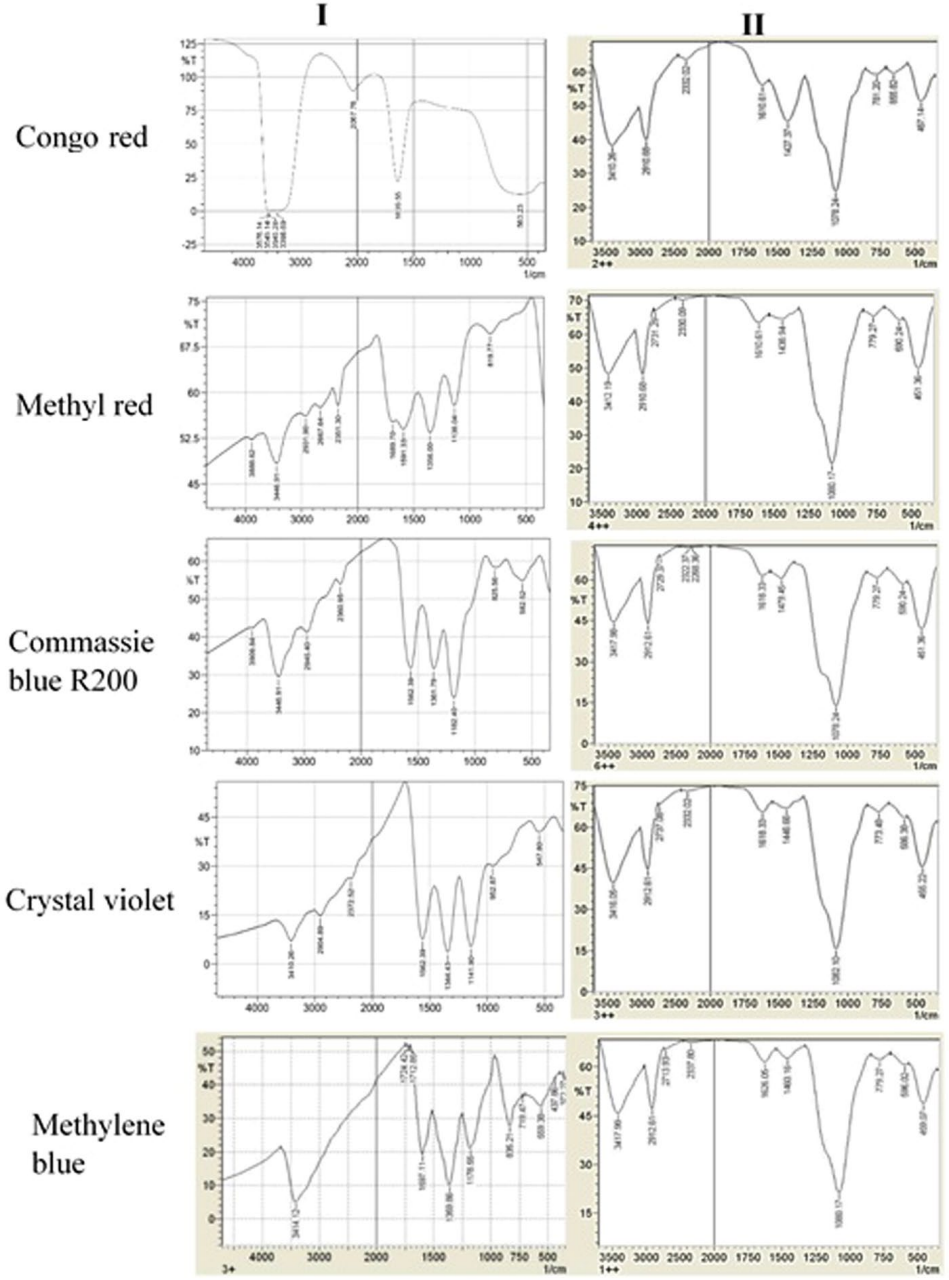
FTIR Spectra of Congo red dye, Methyl red, commassie blue R200, crystal violet and Methylene blue before (I) and after decolorization (II) by the LPO modified silica NPs in the presence of 3.2 mM H_2_O_2_ after incubation for 24 h.

FTIR spectra of the five synthetic dyes (Congo red, crystal violet, methylene blue, methyl red and commassie blue R250) by the LPO modified silica NPs in the presence of 3.2 mM H_2_O_2_ and after incubation for 24 h are shown in the Fig. 4. The IR spectra of the decolorized dyes displayed a disappearance of all bands corresponding to the used dyes and showed all peaks of the LPO modified silica NPs, besides appear new bands in comparing to the bands of the control (IR of synthetic dyes), which confirms the completely decolorization and degradation of Congo red, crystal violet, methylene blue, methyl red and commassie blue R250.

### Cytotoxicity assay of the LPO modified silica NPs

**Figure 5:**
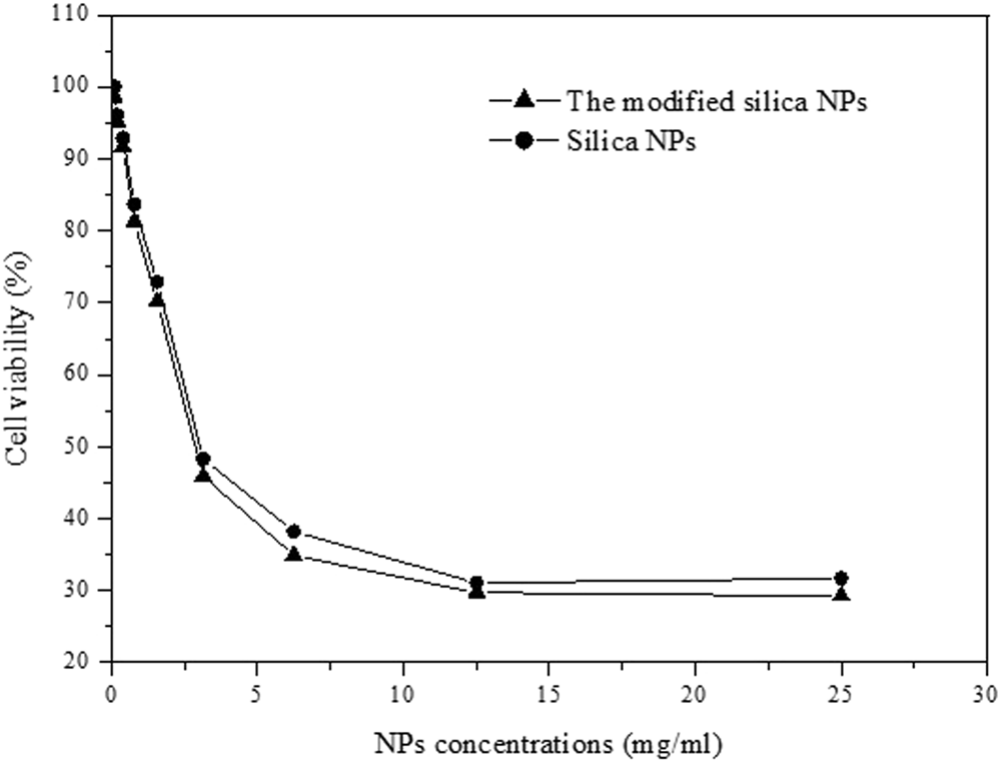
*In vitro* cells viability assay against different concentrations of silica NPs and the LPO modified silica NPs using fibroblast cells.

Cytotoxicity patterns of the silica NPs, and the LPO modified silica NPs were checked on human fibroblast normal cells using neutral red assay protocol in comparison with free LPO and 1 mM H_2_O_2_ after incubation for 48 h. Beginning with 25 mg/ml of the NPs concentrations, the IC_50_ of both silica NP and the LPO modified silica NPs on fibroblast cells reached 2.8 mg/ml. The maximum inhibition percentage of the used NPs on fibroblast cell was 68.3 and 71.10 at concentration of 25 mg/ml for silica NP and the LPO modified silica NPs, respectively (Fig. 5). However, free LPO at the same concentrations of NP and hydrogen peroxide at concentration of 1 mM did not have any cytotoxic effect on the human fibroblast cells.

### Antibacterial activities of the LPO modified silica NPs

**Figure 6:**
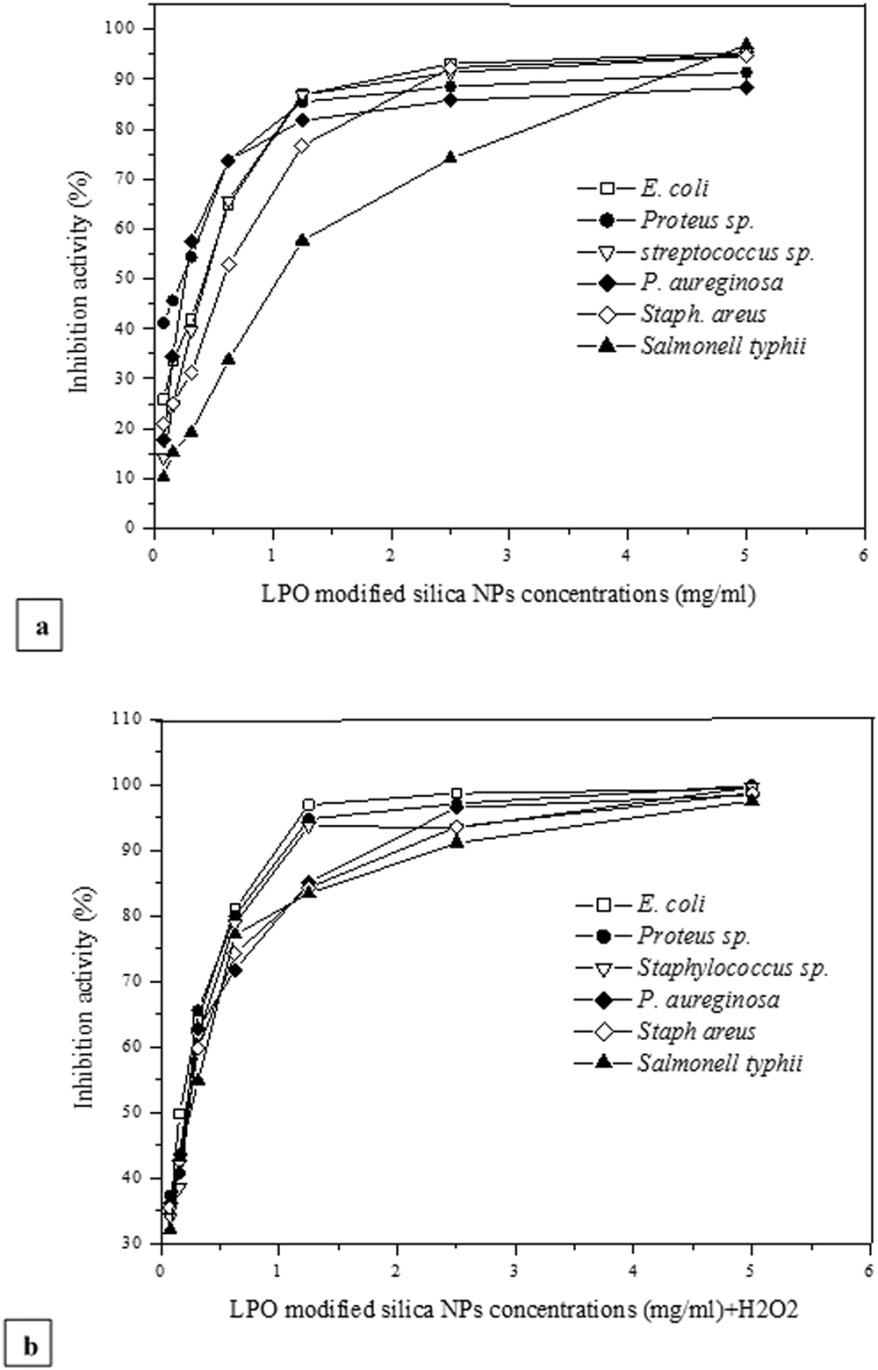
Bactericidal activity of different concentrations the LPO modified silica NPs without H_2_O_2_ (**a**) and in the presence of 1.0 mM H_2_O_2_ (**b**) against *Salmonell typhii*, *Staphylococcus areus*, *Pseudomonas aureginosa*, *E*. *coli*, *Proteus sp*. and *streptococcus sp*. after incubation for 24 h at 37 °C.

Using the plate reader assay protocol, the antibacterial activities of the LPO modified silica NPs and silica NPs were quantified against antibiotic resistant bacterial strains; *Salmonell typhii*, *Staphylococcus areus*, *Pseudomonas aureginosa*, *E*. *coli*, *Proteus sp*. and *streptococcus sp*. Generally, beginning with 5.0 mg/ml of the NPs, *Salmonell typhii*, *E*. *coli* and *streptococcus sp* were found to be the more sensitive strains toward the LPO modified silica NPs treatment with inhibition percentages; 96.88, 95.35 and 94.75, respectively (Fig. 6a). On the other hand, the lowest IC_50_ values were recorded in *Proteus sp*., *E*. *coli*, *Staphylococcus areus* with concentrations of 0.3, 0.4 and 0.625, respectively. Although *Salmonella typhii* was the most sensitive strain toward NPs treatment at the maximum concentration, it showed the highest IC_50_ value; 1.2 mg/ml (Fig. 6a). Additionally, in the presence of 1 mM H_2_O_2_, the LPO modified silica NPs showed a significant antibacterial activity against the same strains as shown in Fig. 6b with a potent efficiency to kill around 95–100% of all bacterial strains at concentrations of 2.5 and 5.0 mg/ml.

**Figure 7:**
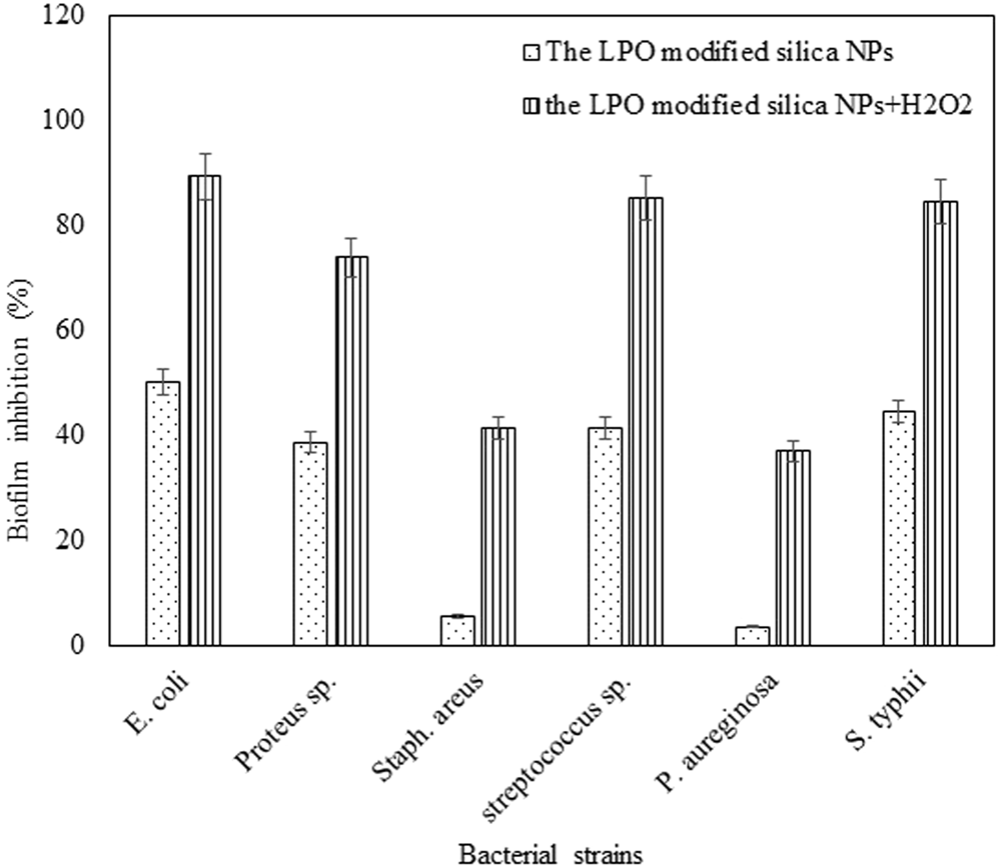
Effect of the LPO modified silica NPs in the presence and absence of 1.0 mM H_2_O_2_ on the bacterial biofilm of *Salmonell typhii*, *Staphylococcus areus*, *Pseudomonas aureginosa*, *E*. *coli*, *Proteus sp*. and *streptococcus sp*. after incubation for 24 h at 37 °C.

Pathogenic bacterial biofilms are associated with persistent infection due to their high resistance to diverse antibiotics. In the present study, the anti-biofilms abilities of the LPO modified silica NPs against all used bacterial pathogens ranged from 49.9–3.6%. *E*. *coli* was the most sensitive bacterial strain (49.9%) to the treatment followed by *Salmonella typhii* (44.7%) and *streptococcus sp*. (41.3%), then *Proteus sp*. (38.67). The biofilm inhibition percentages of both *pseudomonas aureginosa* and *staphylococcus areus* had the lowest values; 3.6 and 5.4, respectively as shown in Fig. 7. However, the LPO modified silica NPs showed a significant anti-biofilm activity in the presence of 1 mM H_2_O_2_ (Fig. 7) with activity around 74–89% against *Proteus sp*., *Salmonella typhii*, *streptococcus sp*. and *E*. *coli*. While free LPO and silica NPs (at the same concentrations) as well as 1 mM H_2_O_2_ failed to exhibit any antibacterial effect or inhibit biofilm of any bacterial strain.

## Discussion

LPO is a monomeric heme containing glycoprotein bound calcium- and iron^33^. LPO is translated as preproprotein of 612 amino acids that contain the N-terminal peptide of about 20 residues and a pro-peptide of about 100 to 300 residues and a catalytic domain. The amino acids sequence of mature bovine LPO contains 15 cysteins that form 7 disulfide bonds; Cys6- Cys167, Cys15-Cys28, Cys129-Cys139, Cys133-Cys157, Cys237-Cys248, Cys456-Cys513, and Cys554-Cys579, while the remaining Cys441 exists as a free cysteine^34^. Bovine LPO is a highly cationic protein with a *pI* of 9.6 and this cationicity is one of the major structural properties of LPO that make LPO has significant roles such as biological activities and antimicrobial potency^33,35,36^. Interaction between protein and NPs considers the origin of NPs bio-reactivity that leads to the formation of NPs-protein complex called corona. Protein corona can affect the conformational changes of the adsorbed proteins that may affect the total bio-reactivity of the NPs. Moreover, the NPs surface can change the structure of the adsorbed protein that leads to modify the function of the protein^22^. In the present study, we revealed that the adsorption of LPO on the surface of silica NPs influences the formation of the modified SiO2-LPO complex which increases retention of LPO, hinting to its usage as an antimicrobial agent for managing the oral bacterial infections.

To that end, the prepared silica NPs were characterized by the FTIR that showed a common band for O-H stretching, bands of H-O-H…H stretching, and bands of SiO-H…H_2_O stretching. However, the broad and the shoulder bands were assigned to asymmetric stretching vibrations of the Si-O-Si for TO and LO modes. The mechanisms of adsorption of proteins on the NPs surface are still not clearly understood. The entropy-driven binding mechanism is one of the mechanisms that is involved in the interaction of proteins with NPs by the release of bound H_2_O from the NPs surface. The reduction in the entropy of adsorbed proteins is less than the rise in entropy of released H_2_O molecules and the conformational of the adsorbed proteins usually does not change^37^. Mesoporous silica NPs are considered one of the most suitable and applied NPs for adsorption and immobilization of enzymes as a result of their specific features such as resistance to hydrothermal conditions, high surface area, and porous structure^38^. Our current research indicated that LPO interacted with porous silica NPs in the presence of phosphate buffer, pH 7.0 to form SiO_2_-LPO nanocombination which separated by centrifugation. The FTIR spectra of the characterized particles revealed shifting in peaks of silica NPs and appearance of another two peaks corresponding to C-H and CH_2_ groups. In addition, LPO interacted with silica NPs to form a hard corona as a result of many reasons such as the S-H group of cysteine residues that are present in the LPO structure as a linker for attachment of LPO on the silica NPs surface. Additionally to high cationicity of LPO which increases its binding to anionic silica NPs, the LPO structure contains basic amino acids with positive charges such as Arg and Lys residues. All of these results are supported by the hypotheses that the surface of electronegative silica NPs electrostatically interacted with LPO. These findings are also supported by Peng *et al*.^39^ They demonstrated that the magnetic NPs had the ability to adsorb proteins according to their surface affinity and charge which influence their effective binding to opposite biomolecules. Furthermore, Saptarshi *et al*.^22^ revealed that the formation of NPs-protein corona is a process that depends on many factors including the interacting proteins and the medium as well as characteristics of the NPs surface such as functional groups and hydrophobicity. On the other hand, numerous investigations have been conducted to test the safety levels of LPO on mammalian cell lines. Hänström *et al*.^40^ investigated the toxicity of several mixtures of LPO-SCN-H_2_O_2_ on human epidermoid carcinoma of the cervix cell line (HeLa), a Chinese hamster ovary cell line (CHO) and human gingival fibroblasts. They described that there were no significant cytotoxic effects of the numerous test mixtures. In addition, the mode of action of silica NPs toxicity was established to be size, dose, and cell type dependent^41^. Our results also indicated that LPO coated silica NPs have a low cytotoxic effect on mammalian fibroblast cells at moderate concentration with an IC_50_ of 2.8 mg/ml. In addition, Abu-Serie and El-Fakharany^42^ demonstrated that coated or loaded LPO-loaded to chitosan NPs with LF was found to be safe on normal cells.

**Figure 8:**
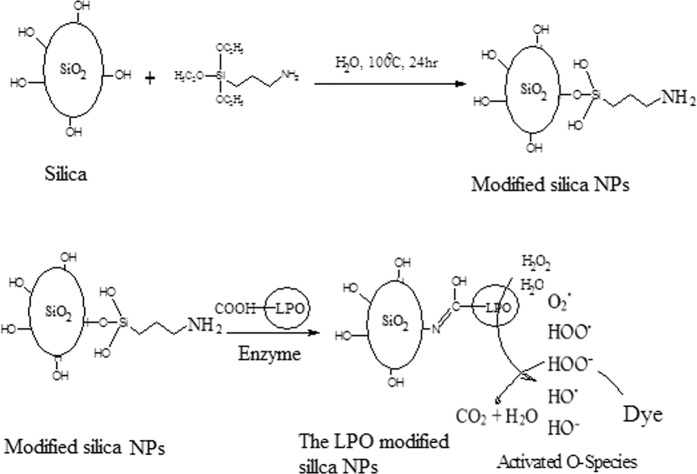
Proposed reaction modes of preparation and decolorization efficiency of the LPO modified silica NPs. Silica NPs were coupled with 3-aminopropyl triethoxysilane to produce the modified silica NPs which interact with LPO through formation of covalent (amide) bond to form the LPO modified silica NPs. The LPO modified silica NPs interact with water and/or H_2_O_2_ in media generating the activated O-species which consequently lead to the direct oxidation of the synthetic dyes. The oxidizing environment leads to complete decolorization and mineralization of the synthetic dyes.

Concerning dye decolorizing abilities, our results indicated that the free LPO enzyme failed to decolorize the synthetic dyes as well as the free silica NPs. Free silica NPs showed decolorization efficiency reached 23% for methylene blue only. However, the LPO modified silica NPs has the ability to decolorize the synthetic dyes (Congo red, Crystal violet, Methylene blue, Methyle red and commassie blue R200) at a high concentration of 500 mg/l in the presence or absence of hydrogen peroxide with decolorization reaching 99% for commassie blue R200. The synthetic dyes under our investigation show Commassie blue R250> Crystal violet> Congo red> Methyl red> methylene blue decolorization pattern using the LPO modified silica NPs in the presence of H_2_O_2_, while in absence of H_2_O_2_ show Methyl red> Congo red> Comassie blue R250> Crystal violet> methylene blue decolorization pattern. The degradation of the synthetic dyes may be due to the modified LPO silica NPs interacting with H_2_O_2_ or water and forming activated O-species. The modified LPO silica NPs have a high oxidation potential activity which leads to the formation of the activated O-species (O_2_^•^, HOO^•^, HOO^−^, HO^•^, and HO^•^) which consequently lead to the direct oxidation of the synthetic dyes. The formed activated O-species, including hydroxyl radicals are an extremely strong and non-selective oxidants^43,44^ that leads to the partial or complete decolorization and degradation of the tested synthetic dyes (Fig. 8). Herein, we demonstrated that the modified LPO silica NPs also showed positive results in effectively killing antibiotic resistant bacterial strains; *Salmonell typhii*, *Staphylococcus areus*, *Pseudomonas aureginosa*, *E*. *coli*, *Proteus sp*. and *streptococcus sp*. and effectively preventing *E*. *coli*, *Salmonella typhii*, *Proteus sp*. and *streptococcus sp*. from forming biofilms with sensitive percentages in the presence of H_2_O_2_. LPO exert its antimicrobial activity through oxidoreductases action including formation of reactive compounds such as hypothiocyanite (OSCN−) or hypoiodite (OI−) which selectively react with sulfhydryl groups of microbial proteins and enzymes leading to inhibition of microbial growth. Therefore, the antibacterial activity of LPO depends on its ability to catalyze production of SCN− or halogens (I−, Cl−, Br−) in the presence of H_2_O_2_. While in the present study, antibacterial action of the LPO modified silica NPs might be explained as a result of creating the activated O-species in the media, especially hydroxyl radicals that effectively kill microbial pathogens^45,46^. Due to non-selectively of the activated O-species, they react with molecules in their instant location at a high rate leading oxidative damage, such as DNA oxidation and lipid peroxidation^47^.

Hence, this novel nanoformulation and stable complex (LPO-silica NPs) of the purified LPO from bovine skimmed milk by coating to silica NPs revealed a potent degradation and decolorization of five synthetic dyes. These positive actions of LPO-silica NPs exerted without high risk on normal cells, besides a significant antibacterial activity against multi-resistant bacterial strains. These findings occur at low concentration of the LPO-silica nanocombination against high concentration of synthetic dyes in comparison with free LPO and silica NPs. These results provide a support for future applications of coating LPO to others NPs as potential antimicrobial agents without risk on normal cells. Furthermore, many food spoilage or pathogenic bacteria can attach to the surfaces of food products forming a biofilm besides, there are different varieties of synthetic dyes used in the food industry. So, the prepared modified SiO_2_-LPO complex can be used as an effective agent for preventing these problems through its ability to remove the toxic dyes and its potent effect against multidrug resistant pathogenic bacteria.

## Conclusion

The silica NPs has a promising surface structure to be modified with protein to prepare a new potent nanocombinations. Here, LPO was absorbed on the surface of silica NPs to produce the LPO modified silica NPs. The formed SiO_2_-LPO complex was characterized by FT-IR and SEM. The findings of the antibacterial and anti-biofilm activities revealed that the potent effect of the LPO modified silica NPs against resistant bacteria was significantly enhanced by the active O-species as a result of the interaction of the modified NPs with H_2_O_2_ or water in the media. Furthermore, the LPO modified silica NPs has the potent degradation efficiency to the tested synthetic dyes at a concentration of 500 mg/l. These findings require further investigations *in vivo* for use the LPO modified silica NPs as a promising candidate in both medicine and food industry to control the bacterial infection and remove toxic dyes.

## Materials and Methods

### Purification of bovine lactoperoxidase

Bovine skimmed milk was prepared from raw milk as described previously by El-Fakharany *et al*.^48,49^. The pH of skimmed milk was adjusted to 7.0 by adding an equal volume of 0.2 M phosphate, pH 7.0 and applied to CM-Sephadex C50 column pre-equilibrated with 50 mM phosphate, pH 7.0. To remove unbound proteins, column was washed with same buffer and LPO was eluted with gradient of NaCl (0.0 to 1.0 M) in 50 mM phosphate, pH 7.0 at flow rate of 0.5 ml/min and fraction size of 1.0 ml/fraction using AKTA prime plus FPLC (GE Health care, Sweden). After collection and concentration of fractions containing LPO by centricon with cut off 50 kDa, 50 mg protein was applied to Sephacryl S-100 column (16 × 60, GE Health care, Sweden) pre-equilibrated with 50 mM phosphate, pH 7.0. Purified LPO was eluted with 50 mM phosphate, pH 7.0 containing 0.15 M NaCl at a flow rate of 0.7 ml/min and fraction size of 3 ml. The fractions containing LPO were concentrated and desalted by centricon with cut off 50 kDa. The LPO purity was confirmed by 12% SDS-PAGE according to the method of Laemmli^50^ and native zymogram of LPO was carried out by using 15 mM guaiacol as a substrate in the presence of 3.3 mM H_2_O_2_ according to methods of Chance and Maehly^51^ and El-Fakharany *et al*.^52^.

### Determination of lactoperoxidase activity

Lactoperoxidase activity was assayed and determined by the procedure of Shindler^53^ with some modification. This method is based on measurement of oxidized ABTS as a result of the oxidizing ABTS as a chromogenic substrate in the presence of hydrogen peroxide at 412 nm using coefficient; 32400 M-1 cm^−1^ according to Özdemir and Uguz^54^. The reaction was carried out at room temperature and initial rates were calculated from time-dependent absorbance changes that reflected enzyme activity. The reaction mixture was carried out in 3 ml containing 0.1 M phosphate borate buffer, pH 6.0, 1 mM ABTS, 3.3 mM H_2_O_2_ and 50 μl of LPO solution. The reaction was started by addition of 50 μl from 3.3 mM H_2_O_2_ to the reaction mixture. The absorbance was measured at 412 nm as a function of times every 15 seconds within 3 min.

### Preparation of Silica nanoparticles

About 2.6 g of CTAB was added to 69 ml of deionized water and stirred to give a clear solution. After that, 8.2 ml of ethanol was added to the surfactant solution. Then, 9.3 ml of an aqueous solution of sodium silicate was added to the surfactant solution to yield a white suspension. The suspension left for 24 h to form two separated clear phases, get rid the upper phase, adding absolute alcohol to the remained phase to form a white precipitate. After that, 1 ml of this suspension was added to a solution of 30 ml methanol, 40 ml double distilled water and 0.5 ml ammonia hydroxide then stirred for 0.5 h, finally 1.5 ml tetramethyl orthosilicate (TMOS) was added to form a milky solution, this solution stirred for 1 hr. the precipitate was separated by centrifuge, dried at 100 °C for 24 h and ignite at 600 °C for 4 h.

### LPO modification of silica nanoparticles

For modification of silica NPs by protein, firstly, silica NPs were reacted with aqueous solution of 3-aminopropyl triethoxysilane at 100 °C for 24 h, then the modified silica NPs were separated by centrifugation, washed 3 times with ethanol and water and dried at 70 °C. Finally, 1 g of the modified silica NPs were added to 25 mg of lyophilized LPO resuspended in 50 ml of 50 mM phosphate buffer, pH 7.0, and stirred for 2 h. After that, the LPO modified silica NPs were separated by centrifugation at 5000 rpm for 10 min and preserved at −4 °C until use.

### Characterization of the prepared modified nanoparticles

The purified LPO concentration in the supernatant during the modified NPs preparation was assayed according to the method of Bradford^55^ and LPO coating efficiency to NPs was calculated as follow: coating (%) = [(X − Y)/X] × 100. Where X is the total concentration of the purified LPO, Y is the concentration of free LPO in the supernatant. Measuring of NP size was done using scanning electron microscopy (SEM) (JSM-636 OLA, Jeol, Japan.). Also, morphological analysis of the silica NP and the LPO modified NPs was characterized SEM at 20 kV. Fourier transmission infra-red spectroscopy (FT-IR) (8400 s, Shimadzu, Japan) covered the range from 400–4000 cm^−1^ was used to detect IR spectra of solid samples using KBr disc method.

### Assaying of synthetic dyes decolorization

Synthetic dyes were prepared by dissolving Congo red, crystal violet, methylene blue, methyl red and commassie blue R250 in 0.1 M phosphate buffer, pH 7.0. Decolorization test was performed in a final volume of 2.0 ml volume by adding 2.5 mg/ml of the LPO modified silica NPs with LPO or free silica NP to each dye solution at concentration of 500 mg/l and with or without adding 3.2 mM H_2_O_2_, followed by incubation of the reaction mixture at room temperature for 6 hr. The percentage of dye decolorization by the LPO modified silica NPs after adding H_2_O_2_ was measured by monitoring absorbance of the taken samples each 1 h using a UV-vis spectrophotometer and the absorbance was measured at 485 nm for congo red, 584 nm for crystal violet, 662 nm for methylene blue, 410 nm for methyl red and 595 nm for coomassie blue. The percentage of decolorization was calculated according to the following formula: Decolorization (%) = [(Ci − Ct)/Ci] × 100, where, Ci is initial absorbance of the dye and Ct is absorbance of the dye along the time.

### Cytotoxicity assay of the LPO modified silica NPs

The safety pattern of the LPO modified silica NPs was evaluated on normal and non-cancerous fibroblast cells in comparison with silica NPs and 1 mM H_2_O_2_. Briefly, about 100 µl of each of serially diluted compound at concentrations ranged from 0.1 mg/ml to 25 mg/ml was incubated with pre-cultured (6.0 × 10^4^ cells/ml) cells in 96-wells tissue culture plates. After 48 h from incubation in suitable conditions, the cellular cytotoxic effects were quantified using neutral red assay protocol^56^. The half-maximal inhibitory concentration (IC_50_) values of silica NP and the LPO modified silica NPs that cause 50% cell viability were estimated.

### Antibacterial activity of the LPO modified silica NPs

Antibacterial activity of the LPO modified silica NPs against the resistant bacterial strains (*Salmonell typhii*, *Staphylococcus areus*, *Pseudomonas aureginosa*, *E*. *coli*, *Proteus sp*. and *streptococcus sp*.) at concentrations of 0.078–5.0 mg/ml was evaluated by a microplate reader assay according to our previously mentioned protocols^57^. The inhibition percentage of the LPO modified silica NPs in the presence and absence of 1 mM H_2_O_2_ was calculated according to the following equation: Inhibition percentage (%) = [A − A1/A0] × 100, Where, A is the absorbance of the treated group, A1 is the absorbance of the blank (NPs delutions) and A0 is the absorbance of the control group. The antibacterial activity of SiO2-LPO complex was performed in comparison with free silica NPs and free LPO as well as 1 mM H_2_O_2_.

### The inhibitory effects of the LPO modified silica NPs on biofilm formation of resistant pathogenic bacteria

Biofilm formation assay was performed by the tissue culture plate (TCP) method^58^. Bacterial strains were inoculated from fresh agar plates in tryticase soy broth (TSB) media and incubated for overnight at 37 °C. Each bacterial strain was standardized to contain about 10^6^ CFU/ml in trypticase soy broth (TSB). For designing the experiment, equal proportions of the standardized microbial suspensions were inoculated in triplicates in sterile, polystyrene, 96 well tissue culture plates. After incubation for 24 h at 37 °C, supernatant of each well was removed and washed three times with 200 µL PBS, pH 7.2 to remove free-floating ‘planktonic’ bacteria. Then 200 µL aliquots of the diluted LPO modified silica NPs at a concentration of 0.19 mg/ml were added to adherent bacterial biofilms in the presence and absence of 1 mM H_2_O_2_ in comparison with silica NPs and 1 mM H_2_O_2_ and plates were incubated at 37 °C for 24 h. Blank controls (medium without inoculum) and positive control (inoculated medium without treatment) were included. The adherent bacterial films were stained with 200 µL of a 0.1% (w/v) aqueous solution of crystal violet (CV) for 5 min. The biofilm formation was quantified by solubilization of the CV stain in 30% glacial acetic acid and the dye concentrations were quantified by microplate reader at a wavelength of 492 nm. The mean absorbance obtained from the medium control well was deducted from the test absorbance values^58^.

### Statistical analysis

The statistical significance was performed using student’s *t*-test and McNemar’s test. The results were expressed as mean ± S error of three different experiments measurements. A (p)-values of <0.05 were considered statistically significant.

## Supplementary information

figure S1

## Data Availability

All data generated or analyzed during this study are included in this published article.
