# Supplementary material for: Preparation and characterization of novel nanocombination of bovine lactoperoxidase with Dye Decolorizing and anti-bacterial activity
Source: Sci Rep. 2019 Jun 12;9:8530. doi: 10.1038/s41598-019-44961-2 (PMC6561912; doi:10.1038/s41598-019-44961-2)
Supplement: Supplementary file 1 — figure S1 [file 41598_2019_44961_MOESM1_ESM.pdf]

Preparation and characterization of novel nanocombination of bovine lactoperoxidase with Dye Decolorizing and anti-bacterial activity

Esmail M. EL-Fakharany[1], Ahmed I. Abd-Elhamid[2], Nehal M. El-Deeb[3].

[1]Protein Research Department, Genetic Engineering and Biotechnology Research Institute (GEBRI), City for Scientific Research and Technology Applications (SRTA-City), New Borg EL Arab 21934, Alexandria, Egypt.

[2]Composites and Nanostructured Materials Research Department, Advanced Technology and New Materials Research Institute, City of Scientific Research and Technological Applications (SRTA-City), New Borg EL Arab 21934, Alexandria, Egypt.

[3]Biopharmacetical Product Research Department, Genetic Engineering and Biotechnology Research Institute (GEBRI), City of Scientific Research and Technological Applications (SRTA-City), New Borg EL Arab 21934, Alexandria, Egypt.

Correspondence and requests for materials should be addressed to E.M.E. (email: esmailelfakharany@yahoo.co.uk)

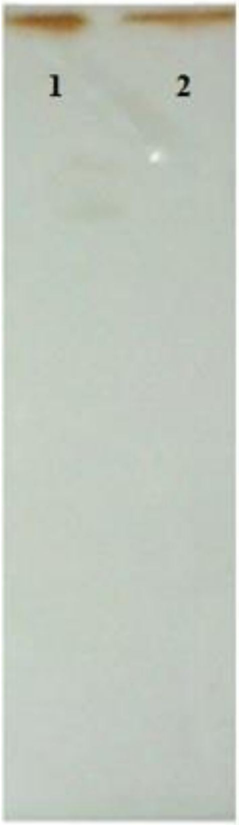

Figure S1. Native zymogram of LPO. Lane 1 is skimmed bovine milk and lane 2 is purified LPO.
